# In Vitro Optical and Physical Stability of Resin Composite Materials with Different Filler Characteristics

**DOI:** 10.3390/polym15092121

**Published:** 2023-04-29

**Authors:** Md Sofiqul Islam, Mohannad Nassar, Mohamed Ahmed Elsayed, Dania Burhan Jameel, Thana Tariq Ahmad, Muhammed Mustahsen Rahman

**Affiliations:** 1 RAK College of Dental Sciences, RAK Medical and Health Sciences University, Ras Al-Khaimah P.O. Box 12973, United Arab Emirates; mohamed.elsayed@rakmhsu.ac.ae (M.A.E.); mustahsen@rakmhsu.ac.ae (M.M.R.); 2 Department of Preventive and Restorative Dentistry, College of Dental Medicine, University of Sharjah, Sharjah P.O. Box 27272, United Arab Emirates; minassar@sharjah.ac.ae; 3 Department of Endodontics, Faculty of Dentistry, Assiut University, Assiut 71515, Egypt

**Keywords:** hardness, water sorption, solubility, color stability, resin composite

## Abstract

The objective of this study was to compare the physical and optical stability of resin composite materials with different filler characteristics. Ninety-six resin composite blocks (6 mm × 6 mm × 2 mm) were prepared using four different types of resin composite, divided into four groups. Specimens from the same material were randomly divided into four groups (*n* = 6) and allocated for Vickers hardness (VH), water sorption, solubility (WS/SL), and staining and aging challenges tests. One-way ANOVA showed significant differences in microhardness (*p* = 0.0001), WS (*p* = 0.0001), and SL (*p* = 0.003) among the tested groups. Beautifil II LS recorded the highest hardness, and CharmFil^®^Flow had the lowest value. Beautifil Injectable X and II LS showed negative WS, whereas the other groups had positive values. All groups showed positive SL. Repeated measures ANOVA showed significant color parameter alteration in the tested groups (*p* = 0.0001). All groups showed significant color shifting after one week of the staining challenge. Repeated measures ANOVA showed a significant color parameter (*p* = 0.0001) and weight (*p* = 0.001) alteration after the aging challenge. The optical and physical stability of resin composites may vary according to filler characteristics. Clinicians should choose the composite as per the desired outcome.

## 1. Introduction

Resin composite is a mainstay restorative material used in routine dental practice to restore carious or lost tooth structure [1]. Resin composite restorative materials have superior aesthetic and functional properties over traditional dental amalgam. One of the main advantages of resin composite is its ability to match the natural color of teeth. This makes it an ideal choice for filling cavities in visible areas of the mouth. The material is available in a variety of shades and can be custom blended to match the color of the patient’s natural teeth. Additionally, resin composite can be used to reshape teeth or close gaps between them, further enhancing the appearance of a patient’s smile [2].

Bonding to tooth structure is another desired property offered by resin composite. Unlike traditional metal fillings, which rely on mechanical retention to stay in place, resin composite forms micromechanical and chemical bonding with the tooth, which results in strengthening the tooth and reducing the risk of further decay or damage. Resin composite is also a durable material that can withstand pressure of biting and chewing. While it may not be as strong as metal fillings, resin composite is strong enough to handle the normal stresses placed on teeth during daily use. Furthermore, the material can be easily repaired intraorally or replaced if needed. Since their introduction in the market, rapid development in their physical and aesthetic properties has been accomplished [3]. The use of resin composite was once limited to anterior teeth to fulfill aesthetic demands, and amalgam was the material of choice for areas of heavy occlusal forces. Nowadays, resin composite is universally used as direct and indirect restorative material for posterior and anterior teeth alike [4,5].

Resin composite materials consist of a matrix of organic resin, typically bisphenol A-glycidyl methacrylate (Bis-GMA) or urethane dimethacrylate (UDMA), and inorganic fillers such as silica, zirconia, or glass ceramics. The organic matrix of resin composites is typically made up of monomers, oligomers, and initiators. Monomers are the building blocks of the resin matrix, while oligomers serve as binders to hold the filler particles together. The most commonly used monomers in resin composites are Bis-GMA, UDMA, and triethylene glycol dimethacrylate (TEGDMA). Bis-GMA has a high molecular weight and is one of the most hydrophobic monomers, making it an excellent choice for resin composite matrices. UDMA has lower viscosity than Bis-GMA, which makes it easier to handle, and TEGDMA is often used as a diluent to reduce the viscosity of the matrix. The inorganic fillers in resin composite are typically added to improve the mechanical properties of the material. These fillers can be either spherical or irregular in shape and range in size from nanometers to micrometers. The most commonly used filler in resin composite is silica, which is typically coated with a silane coupling agent to improve the bond between the filler and the resin matrix. Other fillers used in resin composite include zirconia, glass ceramics, and barium aluminate [6].

The composition of these materials plays a crucial role in determining their properties, including their strength, wear resistance, and color stability. The addition of fillers to the resin matrix can improve the material’s strength, stiffness, and wear resistance. However, excessive filler content leads to decreased translucency and increased opacity, which may negatively affect the material’s aesthetic properties. The size, shape, and distribution of the fillers can also affect the material’s properties. Nano-fillers, for example, have been shown to improve the mechanical properties of resin composite due to their small size and high surface area [7].

BIS-GMA, TEGDEMA, and UDMA are the main matrix components in resin composites that have been used since the beginning and have remained unchanged over the past years. On the other hand, filler particle technology has gone through massive research and development to meet the desired properties and improve resin composite materials. The size, type, volume, weight, polymerization, and optical properties of the filler particles were the key areas of research over the past years [8].

Due to their physical properties, nano-hybrid resin composites are currently the most commonly used resin-based restorative material. The filler percentage in a nano-hybrid resin composite was maximized by the use of different-sized filler particles [9]. On the contrary, flowable composite has a reduced filler percentage to increase fluidity and facilitate penetration into small and deep areas of prepared cavities. Despite having better flowability, the lower filler percentage compromises other properties of a flowable composite. Another recently introduced technology is surface pre-reacted glass-ionomer (S-PRG) filler, which has the ability to release several types of ions, including fluoride (F), borate (B), aluminum (Al), sodium (Na), silicate (Si), and strontium (Sr) ions, that have bioactive functions such as enhancing re-mineralization, halting demineralization, acid buffering, and anti-biofilm properties [10].

Despite the admirable aesthetic outcome that can be achieved with resin composite, the optical and physical stability of these materials remains one of the main challenges in restorative dentistry [11]. A major concern that was pointed out in several studies is the aesthetic alteration over time. A retrospective study by Özdaş D et al. compared the color changes of resin composites from different manufacturers after immersion in multiple liquids for 1, 14, or 30 days and showed significant color changes in all tested materials [12]. Another study by El-Rashidy AA et al. reported that color alteration occurs in both single-shade and multi-shade resin composites after artificial aging and following storage in different media, including artificial saliva, tea, and red wine [13].

The deterioration in the physical properties of resin composite also compromises the long-term success of fillings in restored teeth. Resin composite is inherently prone to water sorption and dissolution in oral fluid over time. Alshali et al. evaluated the water sorption and solubility of both the conventional and bulk-fill resin composites after one-year storage in water and artificial saliva; their findings showed that water sorption and solubility occurred in both tested materials [14]. Ozer et al. reported the solubility of a universal resin composite and a silorane-based resin composite after immersion in a mouth rinse or artificial saliva [15]. In addition to resins and fillers, there are several other components in composite materials such as silane coupling agents, initiators, accelerators, and pigments that might also influence the physical and optical properties [16].

The market for dental materials is rapidly evolving, and new materials are being consistently introduced into the market; thus, there remains a continuous need to evaluate and compare the properties of new products [17]. The objectives of this study were to compare the physical and optical stability of resin composite materials with different filler characteristics by means of micro-hardness (MH), water sorption (WS), solubility (SL), color stability, and aging tests. The null hypothesis tested in this study was that there were no differences between the tested materials in terms of micro-hardness, mass stability, or color parameters.

## 2. Materials and Methods

A3 shade resin composite blocks were prepared using a customized silicon index. Resin composite materials were inserted into a 6 mm × 6 mm × 2 mm sized well and condensed well with composite placing instruments. The well was covered and compressed gently with a glass slide to achieve a smooth uniform surface and to remove excess material. Then, the specimens were polymerized using curing light (Paradigm™ DeepCure LED Curing Light, 3M Oral Care, St. Paul, MN, USA). The curing light was applied to each surface of the blocks for a duration according to the manufacturers’ instructions. A total of 96 blocks were prepared using the test materials (*n* = 24/test material) mentioned in Table 1. Specimens from the same material were randomly and equally divided (*n* = 6) to test the hardness by Vickers hardness test equipment (VH), water sorption, and solubility (WS/SL), and subjected to staining and aging challenges.

Six resin composite blocks from each group were allocated for the Vickers surface micro-hardness test. The Vickers hardness number of the surface of each sample was measured using a micro-hardness tester (Wilson^®^ Tukon™ 1102, Buehler, Echterdingen, Germany). The surface indentations were made at three randomly chosen locations around the center of the surface with at least 1 mm separation from adjacent indentations. Measuring the Vickers hardness creates pyramid-shaped indentations on the surface of the material with an applied load-acting force of 50 g (HV 0.1) for 10 s and an angle of 136° between opposite faces. After removing the load, the ratio between the applied load and the real contact area was measured three times using three different magnification settings. Vickers hardness (VH) is calculated by the following equation:

$$\mathrm{VH}\;=\frac{1854.4L}{D2}$$

where L is the applied load (kg), and D is the indentation area (um) [18].

Six resin composite blocks from each group were allocated for the water sorption and solubility tests. The initial weight (*W*1) of the specimens was measured on a precision balance (U.S. Solid 220 × 0.0001 g Analytical Balance, 0.1 mg Lab Balance Digital Precision Scale). Specimens were then immersed into 12 mL of deionized water and stored in an incubator for 7 days at 37 °C. After the incubation period, specimens were blot dried to remove excess water, and the weight was re-measured (*W*2). The degree of water sorption (WS) was calculated using the formula:

$$\mathrm{WS}=\frac{W2-W1}{W1}$$

Then the specimens were air-dried and stored in a closed container for 48 h with silica gel to dehydrate them. The weight in dry condition (*W*3) was recorded, and the solubility (SL) of each specimen was calculated using the formula:

$$\mathrm{SL}=\frac{W1-W3}{W1}$$

Six resin composite blocks from each group were allocated for the staining test. The initial color parameters of the specimen’s lightness (L*), chroma (C*), and hue (H*) were recorded using a digital color chronometer VITA Easyshade^®^ Advance 4.0 (VITA Zahnfabrik, Bad Säckingen, Germany). Then the specimens were immersed individually in 2 mL of staining solution. For the staining solution, 100 mL of boiling water was poured into a glass beaker, and two tea bags were immersed in the water and kept for 10 min. The tea bags were then removed, and the solution was cooled down to room temperature [19]. The color parameters of the specimens were re-evaluated after 7 days of incubation in the staining solution at 37 °C.

Aging tests were performed on the same number of samples as before (*n* = 6) from each group using a thermo-cycling device (Thermocycler THE-1100, SD MECHATRONIK GMBH, Feldkirchen-Westerham, Munich, Germany). The specimens underwent 10,000 thermal cycles between 5 °C and 55 °C with a dwell time of 30 s and a transfer time of 5 s. The color parameter and weight of each specimen were recorded prior to (L*1, C*1, H*1, W1) and after (L*2, C*2, H*2, W2) thermo-cycling.

The data were analyzed using statistical software (SPSS 24.0, SPSS IBM, Armonk, Westchester County, NY, USA). Multiple comparisons of VH, WS, and SL values among the tested groups were performed using one-way ANOVA and Tukey post hoc test. The color parameter and weight alteration by staining and aging challenges were analyzed using Repeated measures ANOVA and paired samples *t*-test.

## 3. Results

**Figure 1:**
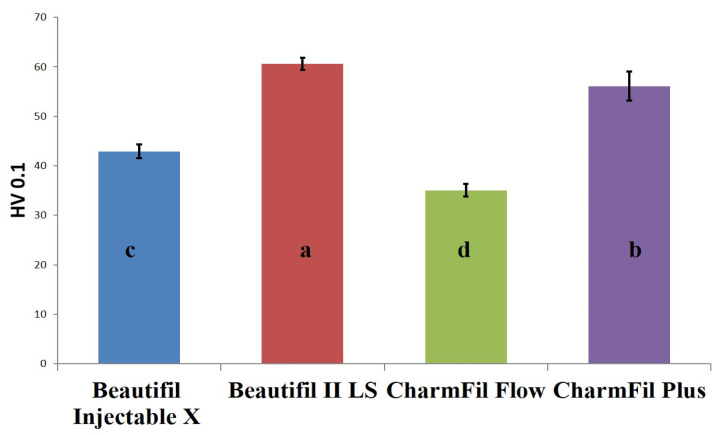
Vickers surface micro-hardness value of experimental groups. Groups with same alphabet are statistically insignificant.

One-way ANOVA showed statistically significant differences in VH (*p* = 0.0001), WS (*p* = 0.0001), and SL (*p* = 0.003) among the tested groups. Group 2 showed the highest hardness among the test groups, followed by group 4, then group 1, and the least was obtained in group 3. The mean hardness value with the standard deviation of each group is shown in Figure 1.

WS results showed that group 1 and group 2 had negative WS values, indicating material weight loss after water immersion. However, the degree of WS between group 1 and group 2 was statistically insignificant (*p* = 0.637). Unlike group 1 and group 2, a positive WS value was observed in groups 3 and 4, indicating that both materials gained weight by absorbing water from the storage media. The degree of WS between group 3 and group 4 was statistically significant (*p* = 0.002). All four groups showed positive SL values after one week of storage in water. Group 3 showed the highest degree of SL, followed by group 2 then group 4, and the least was reported for group 1. The SL of group 3 was statistically significant compared to group 1 (*p* = 0.007); however, it was statistically insignificant compared to group 2 and group 4 (*p* = 0.076). The WS and SL values of the tested groups are shown in Table 2.

Repeated measures ANOVA showed a statistically significant color parameter alteration in the tested groups (*p* = 0.0001). All four groups showed significant color shift after one week of the staining challenge. The elevated C* value and reduction in L* and H* values indicate that the resin composite surfaces were affected by the staining solution and eventually turned darker. Although the same A3 shade was used for all 4 materials, the color parameters L* and C* were significantly different among the tested materials (*p* = 0.0001). This phenomenon reveals the influence of different fillers on the optical properties of resin composite materials. However, after the 1-week staining challenge, differences in L* were statistically insignificant (*p* = 0.114). The H* value, on the other hand, was statistically insignificant among the tested materials (*p* = 0.426) before the staining challenge; however, it became statistically significant after the staining challenge (*p* = 0.001). Color parameters before and after the staining challenge are shown in Table 3.

The results of the aging challenge of resin composite material blocks showed a similar trend compared to the staining challenge in regard to the optical properties. However, unlike the staining challenge, the aging of resin composite blocks significantly elevated the L* and H* values and reduced the C* value; these findings indicate that the resin composite blocks turned whiter and less dense in color. Repeated measures ANOVA showed statistically significant differences in color parameters (*p* = 0.0001) among the tested groups.

The SL values of aged resin composite blocks showed a similar trend to the staining challenge specimens. Repeated measures ANOVA showed a statistically significant difference in the weight of resin composite blocks before and after aging among the tested groups (*p* = 0.001). The weight of flowable composite blocks made from the nano-hybrid and GIOMER was significantly lower than the equal volume of the same conventional composite. The average weight loss of tested GIOMER materials was 3.37%, and that of tested nano-hybrid materials was 5.5%. The color and weight prior to and after the aging challenge are shown in Table 4.

## 4. Discussion

Resin-based restorative materials are part of everyday dental practice and have become the most popular materials to restore decayed or fractured teeth. The increased demand for the clinical use of resin composite has prompted the introduction of several new brands that are available in the market, which also adds to the confusion of the end user on how to accurately select the right material for a certain application [20]. Despite successful results obtained with the use of resin composites, several drawbacks are listed in the literature, which are mainly attributed to their physical and optical properties that deteriorate over time [21,22]. One of the significant drawbacks of resin composites is their susceptibility to polymerization shrinkage. Polymerization shrinkage occurs during the curing process when the material undergoes a chemical reaction, leading to a reduction in the volume of the set product. This shrinkage can cause stress at the composite-tooth interface that might lead to the initiation of micro-leakage, marginal gaps, and secondary caries afterwards. These issues can compromise the longevity of the restoration and lead to clinical failures [23,24]. Another disadvantage of resin composite materials is their vulnerability to wear and degradation over time. Resin composites can undergo surface degradation due to oral fluids, abrasive wear, and thermal cycling. These surface changes are manifested as a loss of gloss and increased surface roughness which compromise esthetics and provide a scaffold for bacterial adhesion and stain uptake, respectively [25]. Furthermore, the use of resin composites for restoring posterior teeth has been reported to fail through bulk and marginal fracture and cuspal deflection, which can compromise the restoration’s durability and longevity in molar and premolar teeth [26]. The current study aimed to investigate some characteristics of four different types of resin composite materials, in which we have found significant differences in VH, color, and mass alteration, WS, SL; thus, these findings require the rejection of the null hypotheses.

The hardness of resin composite materials is an essential characteristic that affects their clinical performance and durability. Hardness is defined as the resistance of a material to indentation or penetration by a harder body. In dentistry, the hardness of a restorative material is often measured using the Knoop or Vickers hardness tests. The hardness of resin composites is influenced by various factors, including the type, size, and distribution of fillers, the degree of conversion of the monomers to polymers, and the presence of any voids or defects within the material. The type and size of fillers used in resin composites have a significant impact on their hardness. Larger fillers tend to increase the hardness of the material due to their greater resistance to deformation, while smaller fillers provide a smoother surface and may improve the polishability of the material. The distribution of fillers within the resin matrix also affects the hardness, with a more uniform distribution leading to higher hardness values. Several studies have investigated the hardness of resin composites and the factors that influence it. A study by Bayne et al. in 1998 evaluated the hardness of different resin composites and found that those materials with higher filler content had greater hardness values [27]. Another study by Ilie et al. in 2013 investigated the effect of curing time and light intensity on the hardness of resin composites and reported that longer curing times and higher light intensities led to increased hardness values [28]. Several other studies reported similar findings related to the hardness of resin composite and its association with the type and load percentage of filler incorporated in resin composite [29,30]. In this study, we used materials with two different types of fillers. Charmfil Flow and Charmfil Plus contain a nano-hybrid type of filler with a filler load of more than 70 wt% (as per the manufacturer). On the other hand, Beautifil injectable X and BeautifilII LS contain S-PRG-type filler with a filler load of approximately 82.9%. Despite the use of the same type and wt% within materials from the same manufacturer, the flowable version of the material of both nano-hybrid and S-PRG filler showed a significantly lower VH value compared to its counterpart of the same brand. A widely accepted explanation for this result is the compositional variation of flowable composites from packable types that significantly lowers the VH value [31]. Another variation that could have affected the results is the degree of conversion in terms of polymerization, which is directly related to the hardness of the set material [32].

In our study, all four resin composite materials, regardless of their filler type and characteristics, turned darker after the staining challenge. Staining of resin composites can occur through various mechanisms, such as extrinsic and intrinsic staining. Extrinsic staining occurs when external agents, such as pigmented foods, drinks, or tobacco, penetrate the surface of the resin composite and become trapped within the material. Intrinsic staining, on the other hand, occurs when pigments are incorporated into the resin matrix during polymerization or are produced through chemical reactions when the material is placed within the oral environment [33]. Filler content also plays a crucial role in determining the stain absorption properties of resin composites. Higher filler content has been shown to decrease stain absorption due to the reduction in the resin matrix surface area. However, excessive filler content can lead to increased surface roughness, which can facilitate the retention and uptake of staining agents [34]. The unpolymerized monomer and the oxygen inhibition layer that exist on the superficial surface of the resin composite are more prone to stain absorption [35]. Surface roughness is another critical factor in the staining of resin composites. Rough surfaces have been shown to promote the adhesion of staining agents, thus enhancing surface discoloration. Studies have reported that polishing the surface of resin-based restorations reduces the degree of stain absorption by creating a smoother surface with fewer retention sites for pigments. A recent study reported that the use of proper finishing and polishing steps and the application of glycerin-based oxygen-inhibiting gel have the potential to minimize stain absorption of both the nano-hybrid and S-PRG-filler-based resin composite materials [19]. In our study, all four types of resin composite turned darker after the staining challenge; however, the aged specimens that did not come in contact with the staining substrate turned lighter after 10,000 thermo-cycling. A possible explanation of this phenomenon could be the unpolymerized monomer on the superficial layer of the resin composite blocks that facilitated the initial chromogen attachment; however, it eventually degraded after thermo-cycling. The weight loss observed after thermo-cycling might be supportive of this speculation. In view of this phenomenon, it is essential that clinicians instruct patients to avoid chromogenic food or beverages immediately after resin composite restoration placement and forewarn them about color alteration that might take place with time.

Water sorption is a critical property of resin composite materials as it can affect their physical and mechanical properties, leading to potential clinical failures. The ability of resin composite materials to absorb water results in dimensional changes, degradation of the matrix and filler, and weakening of the interfacial bond between the composite and the tooth structure. Therefore, understanding the water sorption behavior of resin composites is essential for their optimal use in dental applications and in the development of new materials. The filler content in resin composites can range from 50% to 80% by weight, and it significantly affects the water sorption behavior of the set material. Higher filler content results in lower water sorption due to the increased hydrophobicity of the material. Several studies have investigated the water sorption behavior of resin composites, and the results of these studies have shown that resin composites exhibit varying degrees of water sorption, depending on their composition, filler content, and storage conditions. For instance, resin-based restorative materials with a higher Bis-GMA content tend to absorb more water than those with a higher UDMA or TEGDMA content. Additionally, resin composite materials with larger filler particle sizes have higher water sorption due to the increased surface area available for water absorption. Understanding the factors that affect water sorption and developing strategies to reduce it can improve the performance and longevity of these materials in clinical use [14,36]. In our study, we found that the polymerized conventional nano-hybrid resin composite blocks had an increase in their weight after 7 days of water storage. This phenomenon could be assisted by the comparatively lower filler load in the composition of the material, as described in Table 1. As mentioned earlier, the un-polymerized monomer degradation from the surface of the resin composite block may be responsible for the solubility reported in conventional nano-hybrid composites [37]. Unlike conventional nan-hybrid composites, S-PRG filler is believed to be capable of releasing several types of ions [38]. The initial weight loss after 7 days of water storage shown in groups 1 and 2 could be ascribed to the loss of these ions from the filler component. The solubility characteristics of resin composites are directly correlated with weight loss [39]. The weight loss (W3) of S-PRG filler after dehydration could be associated with the number of ions released during storage in deionized water. Currently, there is a lack of scholarly articles that associate fluoride and other ions release with the mass stability of S-PRG-filler in resin composites to support the hypothetical explanation used in this study.

In general, flowable resin composites have inferior properties compared to conventional composites, thus limiting the clinical use of the former to only certain clinical situations. In our study, the flowable resin composite of both groups showed significantly lower VH values; however, the optical and physical stability was insignificant compared to their conventional counterparts. This finding might pave the way for future research ideas on studying the use of flowable resin composites in various cavities where there is no concern over the lower hardness value of these systems, such as in non-stress-bearing areas of the dentition.

## 5. Conclusions

Within the limitations of the in vitro experiment model, which include, but are not limited to, the use of in vitro experimental conditions contributing to only limited answers to more complex clinical problems, it can be concluded that the physical and optical properties of resin composite materials and their stability over time may vary among different types available in the market. Resin composite materials are prone to stain absorption after polymerization, regardless of filler characteristics. Thus, it is of great importance to accurately assess the different characteristics of newly introduced materials before providing recommendations on their clinical use, and clinicians should select the type of resin composite material as per the desired application and outcome of the restoration and apply an evidence-based practice to improve decision making.

## Figures and Tables

**Table 1:** Group distribution and product detail of experimented materials.

Groups	Tested Materials	Manufacturer	Product Detail
1	Beautifil Injectable X (Shade: A3)	SHOFU INC. Kyoto, JAPAN	Bis-GMA, TEGDMA, Bis- MPEPP, S-PRG filler based on fluoroboroaluminosilicate glass, Polymerization initiator, Pigments and others. Filler load 82.9%
2	Beautifil II LS (Shade: A3)	SHOFU INC. Kyoto, JAPAN	Glass powder, Urethane diacrylate, Bis-MPEPP, Bis-GMA, TEGDMA, Polymerization inhibitor, Pigment and others. Filler load 82.9%
3	CharmFil^®^Flow (Shade: A3)	Dentkist Inc, Gunpo-si, Gyeonggi-do, Korea	Bis-GMA, Barium glass, Triethyleneglycol dimethacrylate, Diurethane dimethacrylate, others. Filler 70% by weight
4	CharmFil^®^Plus (Shade: A3)	Dentkist Inc, Gunpo-si, Gyeonggi-do, Korea	Bis-GMA, Barium glass, Triethyleneglycol dimethacrylate, Diurethane dimethacrylate, others. Filler 70% by weight

**Table 2:** Water sorption and solubility value of each experimental group. Groups with same alphabet are statistically insignificant.

Group	1	2	3	4	*p* Value
WS (/gm)	−0.022 ± 0 01 c	−0.075 ± 0.06 c	0.351 ± 0.18 a	0.181 ± 0.105 b	0.0001
SL (/gm)	0.032 ± 0.013 b	0.090 ± 0.065 a	0.099 ± 0.050 a	0.050 ± 0.047 ab	0.003

**Table 3:** Color parameters of each experimental group before and after the staining. Groups with same alphabet are statistically insignificant.

Group	L*1	L*2	*p* Value	C*1	C*2	*p* Value	H*1	H*2	*p* Value
1	85.0 ± 0.99	63.8 ± 3.91	0.001	28.6 ± 2.33	30.7 ± 2.33	0.019	103.4 ± 4.13	85.2 ± 3.40	0.007
2	86.5 ± 0.14	65.9 ± 1.71	0.001	24.6 ± 1.17	35.8 ± 2.83	0.002	102.9 ± 1.61	84.3 ± 4.01	0.002
3	78.1 ± 1.85	60.1 ± 4.61	0.010	17.0 ± 0.81	26.7 ± 3.07	0.009	105.7 ± 3.14	77.6 ± 1.09	0.001
4	81.4 ± 0.61	64.7 ± 1.01	0.001	19.6 ± 0.72	28.5 ± 5.63	0.062	106.6 ± 4.54	74.9 ± 2.29	0.001
*p* value	0.0001	0.114	-	0.0001	0.024	-	0.426	0.001	-

**Table 4:** Color parameters and weight of each experimental group before and after 10,000 thermo-cycling. Groups with same alphabet are statistically insignificant.

Group	L*1	L*2	*p* Value	C*1	C*2	*p* Value	H*1	H*2	*p* Value	W1 (mg)	W2 (mg)	*p* Value
1	86.1 ± 2.06	100.5 ± 1.0	0.001	27.3 ± 2.67	8.03 ± 2.82	0.001	102.5 ± 3.31	115.8 ± 7.6	0.005	230.3 ± 11.2	223.5 ± 10.1	0.401
2	86.1 ± 1.82	99.6 ± 0.88	0.002	27.2 ± 0.62	14.9 ± 6.56	0.029	101.0 ± 2.9	103.7 ± 3.9	0.384	294.9 ± 10.2	283.5 ± 6.8	0.172
3	79.6 ± 1.82	99.7 ± 0.57	0.001	17.3 ± 0.99	15.2 ± 1.79	0.160	108.8 ± 2.53	112.8 ± 11	0.536	227.1 ± 8.8	215.3 ± 6.7	0.076
4	81.0 ± 3.18	100.1 ± 1.2	0.001	18.4 ± 1.52	14.0 ± 4.91	0.123	110.8 ± 8.67	118.3 ± 10.7	0.089	287.0 ± 10.4	271.4 ± 7.4	0.050
*p* value	0.002	0.492	-	0.0001	0.123	-	0.046	0.002	-	0.0001	0.0001	-

## Data Availability

The data presented in this study are available on request from the corresponding author (M.S.I.).
